# Leveraging Implementation Science for Effective Antimicrobial Resistance Education and Awareness in the WHO AFRO Region

**DOI:** 10.3390/antibiotics14090861

**Published:** 2025-08-27

**Authors:** Walter Fuller, Otridah Kapona, Yahaya Ali Ahmed

**Affiliations:** 1 World Health Organization Regional Office for Africa, Brazzaville P.O. Box 06, Congo; ali-ahmedy@who.int; 2 Totum Solutions Limited, Lusaka P.O. Box 10101, Zambia; kaponaotridah@gmail.com

**Keywords:** antimicrobial resistance, Implementation Science, WHO Africa Region

## Abstract

Inappropriate antimicrobial use (misuse, overuse, underuse, and abuse), often due to a lack of knowledge, is a major factor driving antimicrobial resistance (AMR). Effective education and awareness programs are crucial for addressing this issue. This paper examines how implementation science can improve AMR education and awareness in the World Health Organization (WHO) African Region. This paper discusses the relevance of implementation science frameworks and practical strategies for adapting AMR initiatives to local contexts. By reviewing the literature and case studies, this paper underscores the need for tailored approaches that reflect the region’s unique socio-cultural and healthcare settings. Integrating implementation science into AMR education can promote sustainable behavior change with regard to antimicrobial use, improve healthcare practices, and help combat AMR.

## 1. Introduction

Antimicrobial resistance (AMR) is a major global health threat requiring comprehensive interventions [1]. The inappropriate use of antimicrobials, often due to insufficient knowledge, significantly contributes to AMR. Effective education and awareness programs are crucial for addressing these issues [2]. Despite efforts by the World Health Organization Regional Office for Africa (WHO AFRO) member states, there remains substantial gaps in creating and implementing evidence-based interventions tailored to regional contexts. As healthcare systems face increasing constraints, evidence-based approaches are essential for optimizing healthcare value and improving public well-being.

Implementation science bridges the gap between research and practice by identifying and addressing barriers to effective intervention implementation [3], often by adapting successful strategies from other settings [4]. Key frameworks include the Theoretical Domains Framework (TDF), which identifies barriers and facilitators to behavior change [5]; Consolidated Framework for Implementation Research (CFIR), and Social Cognitive Theory (SCT), which aids in designing health education campaigns by focusing on social influences and self-efficacy (an individual’s belief in their capacity to perform behaviors necessary to achieve desired outcomes).

Implementation science has been successfully leveraged in several global and regional health initiatives. For example, it has been instrumental in advancing equitable vaccine coverage and delivery within national immunization programs, particularly in under-resourced settings [6,7]. It has also provided structured methodologies to guide the design, adaptation, and scale-up of HIV prevention and care interventions targeting vulnerable populations, such as adolescent sexual minority men across diverse global contexts [8]. Furthermore, the WHO and UNICEF have long recognized its value in strengthening the evidence base for the delivery of interventions to control infectious diseases of poverty and, by extension, other social determinants of health [9]. These applications demonstrate how implementation science frameworks can be employed to overcome cultural, systemic, and infrastructural barriers to health education and behavior change.

Africa’s diverse cultural, social, and economic contexts significantly influence the success of AMR interventions [10]. Implementation science can help tailor interventions to these specific factors, including language barriers, cultural diversity, low health literacy, and limited healthcare access [10]. By applying implementation science principles, AMR education and awareness efforts can be more effectively integrated into real-world settings, enhancing their impact. This paper explores how leveraging implementation science can improve AMR education and awareness in the WHO AFRO region.

## 2. Implementation Science Theoretical Frameworks (CFIR and TDF) and Applicability to AMR Education and Awareness

I. The Consolidated Framework for Implementation Research (CFIR) provides a comprehensive framework for understanding the multiple factors influencing implementation outcomes. It identifies five major domains, including the following:

- Intervention characteristics, such as content, format, and delivery of educational materials, can impact the effectiveness of AMR campaigns. A study by Dyar et al. (2017) demonstrated that multifaceted interventions combining educational materials with feedback on prescribing practices were more effective in reducing inappropriate antibiotic use [11].
- Outer setting: External factors such as policies, regulations, and societal norms can influence the success of AMR awareness campaigns. The European Antibiotic Awareness Day (EAAD) campaign leverages policy support, media engagement, and partnerships with healthcare stakeholders to promote responsible antibiotic use in the region [12].
- Inner setting (the structural, socio-cultural, and organizational context in which implementation occurs): Organizational culture, leadership support, and resources within institutions/organizational settings can affect the implementation of AMR education initiatives. A study by Sikkens et al. (2017) demonstrated that a supportive leadership culture, adequate resources, and staff engagement were key factors in the success of stewardship efforts [13].
- Individual characteristics” Knowledge, attitudes, and beliefs of individuals targeted by AMR campaigns can influence their engagement and trigger behavior change [14].
- Process: Steps involved in implementing the intervention, such as planning, execution, and evaluation, have a bearing on the successful implementation and impact of the intervention.

By applying the CFIR to AMR education and awareness programs, stakeholders can assess the readiness of the context, identify potential barriers and facilitators, and tailor interventions to fit the specific needs of the target audience/region.

II. The Theoretical Domains Framework (TDF) provides a comprehensive approach to understanding and addressing behavior change by identifying key determinants and tailoring interventions accordingly. These include the following:

- Knowledge: Inadequate or lack of awareness about AMR and appropriate antimicrobial use can hinder behavior change [15].
- Beliefs about Consequences: Perceptions and apperception of the impact of inappropriate antimicrobial use can affect behavior [16].
- Social Influences: Social norms and peer influence can impact antimicrobial use-related behaviors [17].
- Environmental Context and Resources: Availability of resources and support within healthcare settings and institutions responsible for improving awareness and understanding of AMR through effective communication, education, and training can influence the effectiveness of AMR education and awareness interventions [18].

TDF’s adaptability to various healthcare settings and populations allows it to support global AMR initiatives by targeting behavioral barriers and facilitators to promote sustained change among healthcare professionals, policymakers, and the public.

III. Social Cognitive Theory (SCT): While not a central framework like CFIR or TDF, SCT complements implementation science by providing insights into how behavior change can be achieved and sustained through social and cognitive processes. It can be applied within the broader framework of implementation science to enhance understanding and effectiveness of interventions. SCT emphasizes the reciprocal interactions between individuals and their social environment in shaping behavior. It posits that behavior change is influenced by self-efficacy, observational learning, and social support. In the context of AMR education and awareness, the SCT can guide the development of interventions that strengthen self-efficacy, provide role models for behavior change, and engage influential individuals and organizations to enhance social support and normative behaviors. Modeling behavior through the observation of others can influence behavior change, as demonstrated by Castro-Sánchez et al. (2016) [19]. Belief in one’s ability to perform a behavior (self-efficacy) is a key predictor of behavior change [20]. Additionally, peers, healthcare providers, and community members can influence behavior change [21], and providing positive reinforcement for desired behaviors can encourage behavior change [22].

## 3. Integration of Implementation Science Frameworks for Improved Outcomes

- CFIR and TDF: The CFIR can be used to identify factors that influence implementation success, while the TDF can help in understanding behavior change among individuals involved in the implementation process. In relation to AMR, these two would enable a better understanding of the target audience and tailoring of education and awareness interventions.
- CFIR and SCT: The CFIR can help in identifying organizational factors influencing implementation, while SCT can provide insights into individual behavior change mechanisms.
- TDF and SCT: The TDF can help in identifying behavior change determinants, while SCT can provide a theoretical basis for understanding and predicting behavior change, as a critical factor influencing antimicrobial use, one of the key drivers of AMR.

It is, however, important to note that the effectiveness of a framework depends on the specific context of implementation, the theory being applied, and the goal/s of the intervention.

## 4. Adapting Interventions to Local Contexts

A critical part of implementation science is adapting treatments to local circumstances. The study of techniques that encourage the integration of research findings and evidence-based procedures into healthcare policy and practice is the focus of implementation science. Table 1 lists some major concerns and ways for adapting interventions to local contexts:

## 5. Conclusions

Implementation science offers a critical pathway for advancing the antimicrobial resistance education and awareness agenda, particularly through the development of context-responsive tools and evidence-based strategies that facilitate sustainable behavior change. In the WHO African Region, where socio-anthropological factors significantly influence health behaviors, a participatory implementation approach, one that actively engages key stakeholders throughout the planning, execution, and evaluation phases, can substantially enhance the effectiveness of AMR education and awareness interventions.

By intentionally addressing cultural and structural barriers, tailoring programs to local contexts, building institutional and community-level capacity, and embedding principles of cultural competence, collaboration, adaptability, and continuous monitoring, these interventions can achieve greater resonance and impact. This perspective contributes to the field by integrating and synthesizing multiple implementation science frameworks, an approach still underutilized in the AMR landscape of the region, to propose actionable, evidence-informed strategies for regional adaptation.

Moreover, by linking behavioral science constructs with practical delivery mechanisms, this paper demonstrates how implementation frameworks can be operationalized to improve the reach, relevance, and sustainability of AMR communication efforts. Such strategic integration is essential for achieving long-term containment of AMR and advancing health systems resilience in the African context.

We therefore propose the following priorities to strengthen AMR education in the WHO AFRO region: (1) establish culturally competent, cross-disciplinary implementation teams within national AMR action plans; (2) integrate targeted AMR messaging into existing primary healthcare and community outreach systems; and (3) institutionalize participatory feedback mechanisms to continuously refine education strategies based on real-world insights.

## Figures and Tables

**Table 1:** Thematic clusters of strategies and actions for culturally responsive AMR interventions.

Thematic Cluster	Strategy	Enhanced Action
Cultural Relevance and Community Engagement	Cultural Competence and Diversity	Conduct structured cultural competence training for implementation teams. Form multidisciplinary teams representative of the target community, ensuring inclusion across sectors (human, animal, environment).
	Community Involvement and Co-creation	Use participatory methods (e.g., CBPR, co-design workshops) that effectively engage communities in designing and evaluating HIV prevention programs [23] and peer education programs, which empower individuals to educate peers and reduce risky behaviors in sexual health [24]. Establish community advisory groups and engage local champions to build trust and foster ownership.
Communication and Knowledge Transfer	Contextualized Communication	Tailor messaging to local languages, literacy levels, and cultural norms using appropriate tone, visuals, and formats. Disseminate messages through locally trusted channels such as community radio, SMS, and social media.
	Knowledge Translation	Develop user-friendly learning resources and tools (e.g., guides, infographics, WHO AFRO AMR regional education and awareness webinars-https://who.zoom.us/j/93396381332, accessed on 19 June 2025) Promote continuous learning through mentorship, peer exchange, and integration of AMR topics in existing platforms.
Capacity and Infrastructure	Capacity Building and Resource Planning	Integrate AMR into training curricula for health and allied professionals. Conduct resource mapping to inform budgeting and mobilize support from diverse sources (e.g., public, private, partners).
	Policy and Institutional Alignment	Collaborate with policymakers to translate AMR evidence into supportive policies. Strengthen governance structures, including multisectoral AMR committees and regulatory frameworks.
Adaptability and Sustainability	Adaptive Implementation	Pilot interventions to test feasibility and refine approaches. Tailor delivery methods, messages, and tools to local contexts and preferences.
	Monitoring, Evaluation, and Learning	Develop SMART indicators and integrate real-time feedback tools (e.g., surveys, dashboards). Use findings to adapt and scale interventions, and share lessons across regions.
	Sustainability Planning	Embed AMR interventions into existing health systems and community platforms. Align activities with national AMR plans and foster multisectoral partnerships for long-term impact.

## Data Availability

The information presented in this review is available in open-access journals.
